# Supplementary material for: Effect of cis- and trans-Monounsaturated Fatty Acids on Palmitate Toxicity and on Palmitate-induced Accumulation of Ceramides and Diglycerides
Source: Int J Mol Sci. 2020 Apr 9;21(7):2626. doi: 10.3390/ijms21072626 (PMC7178055; doi:10.3390/ijms21072626)
Supplement: Supplementary file 1 [file ijms-21-02626-s001.pdf]

	Control		Palmitate		Palmitate + Elaidate		Palmitate + Oleate		Palmitate + Vaccenate	
	Mean	*SEM*	*Mean*	*SEM*	*Mean*	*SEM*	*Mean*	*SEM*	*Mean*	*SEM*
	μg/mg protein									
	4 h									
**DG 16:0/16:0**	1.37	0.13	28.73	0.82	6.97	0.61	5.74	0.64	7.55	0.43
**DG 16:0/16:1**	0.42	0.03	1.15	0.07	0.47	0.05	0.34	0.04	0.50	0.03
**DG 16:0/18:0**	0.48	0.05	4.58	0.34	1.23	0.16	0.82	0.11	1.37	0.16
**DG 16:0/18:1**	1.60	0.15	2.89	0.30	6.47	0.75	4.42	0.49	8.52	0.78
**DG 18:0/18:0**	0.04	0.01	0.14	0.02	0.09	0.01	0.06	0.01	0.08	0.01
**DG 18:0/18:1**	0.31	0.04	0.34	0.05	0.52	0.08	0.57	0.08	0.55	0.06
**DG 18:1/18:1**	0.75	0.07	0.64	0.08	1.78	0.21	2.16	0.25	2.17	0.19
	8 h									
**DG 16:0/16:0**	1.45	0.19	68.32	4.93	9.15	0.69	6.36	0.68	10.85	1.00
**DG 16:0/16:1**	0.43	0.04	2.31	0.24	0.56	0.06	0.34	0.04	0.66	0.07
**DG 16:0/18:0**	0.66	0.09	11.49	1.17	1.88	0.25	1.28	0.20	1.95	0.28
**DG 16:0/18:1**	1.48	0.21	5.00	0.72	9.80	1.25	4.65	0.58	12.36	1.56
**DG 18:0/18:0**	0.04	0.00	0.33	0.03	0.08	0.01	0.07	0.01	0.09	0.02
**DG 18:0/18:1**	0.42	0.06	0.80	0.12	0.73	0.10	0.81	0.12	0.77	0.11
**DG 18:1/18:1**	0.72	0.12	0.70	0.12	2.81	0.43	2.02	0.28	3.08	0.43
	16 h									
**DG 16:0/16:0**	2.05	0.24	49.91	2.36	11.44	1.30	5.59	0.47	11.37	1.04
**DG 16:0/16:1**	0.52	0.04	2.91	0.33	0.74	0.09	0.36	0.03	0.81	0.08
**DG 16:0/18:0**	1.11	0.18	9.82	0.85	3.61	0.70	1.39	0.17	2.76	0.44
**DG 16:0/18:1**	2.01	0.26	6.87	1.18	19.81	3.57	5.39	0.63	14.60	1.66
**DG 18:0/18:0**	0.06	0.01	0.39	0.04	0.18	0.04	0.07	0.01	0.17	0.03
**DG 18:0/18:1**	0.51	0.06	0.96	0.15	1.31	0.25	0.90	0.11	0.78	0.14
**DG 18:1/18:1**	0.90	0.11	1.00	0.22	6.96	1.37	2.45	0.41	3.46	0.46
	24 h									
**DG 16:0/16:0**	2.63	0.29	33.33	1.23	10.88	0.57	5.71	0.47	11.05	0.85
**DG 16:0/16:1**	0.71	0.05	2.33	0.13	0.95	0.08	0.41	0.03	0.98	0.08
**DG 16:0/18:0**	1.43	0.19	6.89	0.43	3.15	0.37	1.77	0.23	2.31	0.32
**DG 16:0/18:1**	2.96	0.37	4.58	0.58	15.84	1.64	5.74	0.52	13.22	1.48
**DG 18:0/18:0**	0.12	0.01	0.36	0.03	0.17	0.02	0.15	0.03	0.12	0.01
**DG 18:0/18:1**	1.00	0.16	0.79	0.12	1.35	0.21	1.53	0.20	0.85	0.13
**DG 18:1/18:1**	1.31	0.19	0.57	0.08	5.78	0.74	2.43	0.22	3.21	0.39

Table S1. Diglyceride accumulation. Cells were treated with BSA (control cells) or BSA-conjugated palmitate alone, or with palmitate and one of the unsaturated fatty acids, elaidate, oleate or vaccenate at 250 μM individual concentration for 8 h at 70-80% confluence. The amount of seven major diglyceride species was measured by LC-MS/MS in the washed cell samples prepared after 4, 8, 16 or 24 hour treatments. The detected diglycerides contained the indicated combinations of palmitate (16:0), stearate (18:0), and a monounsaturated fatty acid of 16 (16:1) or 18 carbons (18:1). Data were normalized to the protein content of the samples (μg/mg protein units), and are shown as mean values and SEM of three independent experiments with two parallels.

	Tukey's multiple comparisons test			
		**Mean Diff.**	**95.00% CI. of diff.**	**Significant?**
DG 16:0/16:0	C 4 h vs. P 4 h	-27.36	-32.98 to -21.73	Yes
	C 4 h vs. P + E 4 h	-5.596	-11.22 to 0.02973	No
	C 4 h vs. P + O 4 h	-4.37	-9.995 to 1.256	No
	C 4 h vs. P + V 4 h	-6.174	-11.8 to -0.5478	Yes
	P 4 h vs. P + E 4 h	21.76	16.14 to 27.39	Yes
	P 4 h vs. P + O 4 h	22.99	17.36 to 28.61	Yes
	P 4 h vs. P + V 4 h	21.18	15.56 to 26.81	Yes
	P + E 4 h vs. P + O 4 h	1.226	-4.4 to 6.852	No
	P + E 4 h vs. P + V 4 h	-0.5776	-6.203 to 5.048	No
	P + O 4 h vs. P + V 4 h	-1.804	-7.429 to 3.822	No
	C 8 h vs. P 8 h	-66.86	-89.25 to -44.48	Yes
	C 8 h vs. P + E 8 h	-7.693	-30.08 to 14.69	No
	C 8 h vs. P + O 8 h	-4.909	-27.29 to 17.48	No
	C 8 h vs. P + V 8 h	-9.399	-31.78 to 12.99	No
	P 8 h vs. P + E 8 h	59.17	36.79 to 81.55	Yes
	P 8 h vs. P + O 8 h	61.95	39.57 to 84.34	Yes
	P 8 h vs. P + V 8 h	57.46	35.08 to 79.85	Yes
	P + E 8 h vs. P + O 8 h	2.784	-19.6 to 25.17	No
	P + E 8 h vs. P + V 8 h	-1.706	-24.09 to 20.68	No
	P + O 8 h vs. P + V 8 h	-4.49	-26.87 to 17.89	No
	C 16 h vs. P 16 h	-47.86	-60.68 to -35.04	Yes
	C 16 h vs. P + E 16 h	-9.385	-22.2 to 3.433	No
	C 16 h vs. P + O 16 h	-3.538	-16.36 to 9.28	No
	C 16 h vs. P + V 16 h	-9.312	-22.13 to 3.506	No
	P 16 h vs. P + E 16 h	38.47	25.65 to 51.29	Yes
	P 16 h vs. P + O 16 h	44.32	31.5 to 57.14	Yes
	P 16 h vs. P + V 16 h	38.55	25.73 to 51.36	Yes
	P + E 16 h vs. P + O 16 h	5.847	-6.97 to 18.67	No
	P + E 16 h vs. P + V 16 h	0.07307	-12.74 to 12.89	No
	P + O 16 h vs. P + V 16 h	-5.774	-18.59 to 7.043	No
	C 24 h vs. P 24 h	-30.7	-38.07 to -23.34	Yes
	C 24 h vs. P + E 24 h	-8.256	-15.62 to -0.8894	Yes
	C 24 h vs. P + O 24 h	-3.079	-10.45 to 4.288	No
	C 24 h vs. P + V 24 h	-8.419	-15.79 to -1.053	Yes
	P 24 h vs. P + E 24 h	22.45	15.08 to 29.81	Yes
	P 24 h vs. P + O 24 h	27.62	20.26 to 34.99	Yes
	P 24 h vs. P + V 24 h	22.28	14.92 to 29.65	Yes
	P + E 24 h vs. P + O 24 h	5.177	-2.19 to 12.54	No
	P + E 24 h vs. P + V 24 h	-0.1632	-7.53 to 7.204	No
	P + O 24 h vs. P + V 24 h	-5.34	-12.71 to 2.027	No

DG 16:0/16:1	C 4 h vs. P 4 h	-0.7303	-1.184 to -0.2765	Yes
	C 4 h vs. P + E 4 h	-0.04326	-0.497 to 0.4105	No
	C 4 h vs. P + O 4 h	0.08247	-0.3713 to 0.5362	No
	C 4 h vs. P + V 4 h	-0.07756	-0.5313 to 0.3762	No
	P 4 h vs. P + E 4 h	0.687	0.2333 to 1.141	Yes
	P 4 h vs. P + O 4 h	0.8128	0.359 to 1.267	Yes
	P 4 h vs. P + V 4 h	0.6527	0.199 to 1.106	Yes
	P + E 4 h vs. P + O 4 h	0.1257	-0.328 to 0.5795	No
	P + E 4 h vs. P + V 4 h	-0.0343	-0.4881 to 0.4195	No
	P + O 4 h vs. P + V 4 h	-0.16	-0.6138 to 0.2937	No
	C 8 h vs. P 8 h	-1.881	-3.035 to -0.7263	Yes
	C 8 h vs. P + E 8 h	-0.1268	-1.281 to 1.028	No
	C 8 h vs. P + O 8 h	0.09028	-1.064 to 1.245	No
	C 8 h vs. P + V 8 h	-0.2337	-1.388 to 0.9207	No
	P 8 h vs. P + E 8 h	1.754	0.5995 to 2.908	Yes
	P 8 h vs. P + O 8 h	1.971	0.8166 to 3.125	Yes
	P 8 h vs. P + V 8 h	1.647	0.4926 to 2.801	Yes
	P + E 8 h vs. P + O 8 h	0.217	-0.9374 to 1.372	No
	P + E 8 h vs. P + V 8 h	-0.1069	-1.261 to 1.048	No
	P + O 8 h vs. P + V 8 h	-0.324	-1.478 to 0.8305	No
	C 16 h vs. P 16 h	-2.392	-3.926 to -0.8581	Yes
	C 16 h vs. P + E 16 h	-0.226	-1.76 to 1.308	No
	C 16 h vs. P + O 16 h	0.1579	-1.376 to 1.692	No
	C 16 h vs. P + V 16 h	-0.2898	-1.824 to 1.244	No
	P 16 h vs. P + E 16 h	2.166	0.6321 to 3.7	Yes
	P 16 h vs. P + O 16 h	2.55	1.016 to 4.084	Yes
	P 16 h vs. P + V 16 h	2.102	0.5683 to 3.636	Yes
	P + E 16 h vs. P + O 16 h	0.3839	-1.15 to 1.918	No
	P + E 16 h vs. P + V 16 h	-0.06383	-1.598 to 1.47	No
	P + O 16 h vs. P + V 16 h	-0.4477	-1.982 to 1.086	No
	C 24 h vs. P 24 h	-1.611	-2.424 to -0.797	Yes
	C 24 h vs. P + E 24 h	-0.2304	-1.044 to 0.5831	No
	C 24 h vs. P + O 24 h	0.3028	-0.5108 to 1.116	No
	C 24 h vs. P + V 24 h	-0.2689	-1.082 to 0.5446	No
	P 24 h vs. P + E 24 h	1.38	0.5666 to 2.194	Yes
	P 24 h vs. P + O 24 h	1.913	1.1 to 2.727	Yes
	P 24 h vs. P + V 24 h	1.342	0.5281 to 2.155	Yes
	P + E 24 h vs. P + O 24 h	0.5332	-0.2804 to 1.347	No
	P + E 24 h vs. P + V 24 h	-0.0385	-0.8521 to 0.775	No
	P + O 24 h vs. P + V 24 h	-0.5717	-1.385 to 0.2419	No
DG 16:0/18:0	C 4 h vs. P 4 h	-4.102	-5.948 to -2.256	Yes
	C 4 h vs. P + E 4 h	-0.7455	-2.591 to 1.1	No
	C 4 h vs. P + O 4 h	-0.3383	-2.184 to 1.508	No
	C 4 h vs. P + V 4 h	-0.8923	-2.738 to 0.9536	No
	P 4 h vs. P + E 4 h	3.357	1.511 to 5.203	Yes
	P 4 h vs. P + O 4 h	3.764	1.918 to 5.61	Yes

	P 4 h vs. P + V 4 h	3.21	1.364 to 5.056	Yes
	P + E 4 h vs. P + O 4 h	0.4072	-1.439 to 2.253	No
	P + E 4 h vs. P + V 4 h	-0.1468	-1.993 to 1.699	No
	P + O 4 h vs. P + V 4 h	-0.554	-2.4 to 1.292	No
	C 8 h vs. P 8 h	-10.84	-16.3 to -5.378	Yes
	C 8 h vs. P + E 8 h	-1.227	-6.686 to 4.233	No
	C 8 h vs. P + O 8 h	-0.6268	-6.087 to 4.833	No
	C 8 h vs. P + V 8 h	-1.294	-6.754 to 4.166	No
	P 8 h vs. P + E 8 h	9.611	4.151 to 15.07	Yes
	P 8 h vs. P + O 8 h	10.21	4.751 to 15.67	Yes
	P 8 h vs. P + V 8 h	9.544	4.084 to 15	Yes
	P + E 8 h vs. P + O 8 h	0.5997	-4.86 to 6.059	No
	P + E 8 h vs. P + V 8 h	-0.06727	-5.527 to 5.392	No
	P + O 8 h vs. P + V 8 h	-0.667	-6.127 to 4.793	No
	C 16 h vs. P 16 h	-8.713	-13.99 to -3.435	Yes
	C 16 h vs. P + E 16 h	-2.495	-7.773 to 2.783	No
	C 16 h vs. P + O 16 h	-0.2756	-5.554 to 5.002	No
	C 16 h vs. P + V 16 h	-1.643	-6.921 to 3.635	No
	P 16 h vs. P + E 16 h	6.217	0.9392 to 11.5	Yes
	P 16 h vs. P + O 16 h	8.437	3.159 to 13.72	Yes
	P 16 h vs. P + V 16 h	7.07	1.792 to 12.35	Yes
	P + E 16 h vs. P + O 16 h	2.22	-3.058 to 7.498	No
	P + E 16 h vs. P + V 16 h	0.8525	-4.426 to 6.131	No
	P + O 16 h vs. P + V 16 h	-1.367	-6.645 to 3.911	No
	C 24 h vs. P 24 h	-5.459	-8.58 to -2.338	Yes
	C 24 h vs. P + E 24 h	-1.727	-4.847 to 1.394	No
	C 24 h vs. P + O 24 h	-0.3481	-3.469 to 2.773	No
	C 24 h vs. P + V 24 h	-0.8881	-4.009 to 2.233	No
	P 24 h vs. P + E 24 h	3.732	0.6114 to 6.853	Yes
	P 24 h vs. P + O 24 h	5.111	1.99 to 8.231	Yes
	P 24 h vs. P + V 24 h	4.571	1.45 to 7.691	Yes
	P + E 24 h vs. P + O 24 h	1.379	-1.742 to 4.499	No
	P + E 24 h vs. P + V 24 h	0.8387	-2.282 to 3.959	No
	P + O 24 h vs. P + V 24 h	-0.54	-3.661 to 2.581	No
DG 16:0/18:1	C 4 h vs. P 4 h	-1.295	-6.673 to 4.083	No
	C 4 h vs. P + E 4 h	-4.869	-10.25 to 0.5091	No
	C 4 h vs. P + O 4 h	-2.817	-8.195 to 2.561	No
	C 4 h vs. P + V 4 h	-6.917	-12.29 to -1.539	Yes
	P 4 h vs. P + E 4 h	-3.574	-8.952 to 1.804	No
	P 4 h vs. P + O 4 h	-1.522	-6.9 to 3.856	No
	P 4 h vs. P + V 4 h	-5.622	-11 to -0.2442	Yes
	P + E 4 h vs. P + O 4 h	2.052	-3.326 to 7.43	No
	P + E 4 h vs. P + V 4 h	-2.048	-7.426 to 3.33	No
	P + O 4 h vs. P + V 4 h	-4.1	-9.478 to 1.278	No
	C 8 h vs. P 8 h	-3.523	-13.18 to 6.129	No
	C 8 h vs. P + E 8 h	-8.323	-17.97 to 1.33	No

	C 8 h vs. P + O 8 h	-3.175	-12.83 to 6.478	No
	C 8 h vs. P + V 8 h	-10.88	-20.53 to -1.227	Yes
	P 8 h vs. P + E 8 h	-4.799	-14.45 to 4.853	No
	P 8 h vs. P + O 8 h	0.3487	-9.304 to 10	No
	P 8 h vs. P + V 8 h	-7.356	-17.01 to 2.296	No
	P + E 8 h vs. P + O 8 h	5.148	-4.504 to 14.8	No
	P + E 8 h vs. P + V 8 h	-2.557	-12.21 to 7.095	No
	P + O 8 h vs. P + V 8 h	-7.705	-17.36 to 1.947	No
	C 16 h vs. P 16 h	-4.86	-23.04 to 13.32	No
	C 16 h vs. P + E 16 h	-17.79	-35.97 to 0.3846	No
	C 16 h vs. P + O 16 h	-3.378	-21.56 to 14.8	No
	C 16 h vs. P + V 16 h	-12.59	-30.77 to 5.592	No
	P 16 h vs. P + E 16 h	-12.93	-31.11 to 5.244	No
	P 16 h vs. P + O 16 h	1.482	-16.7 to 19.66	No
	P 16 h vs. P + V 16 h	-7.727	-25.91 to 10.45	No
	P + E 16 h vs. P + O 16 h	14.42	-3.763 to 32.6	No
	P + E 16 h vs. P + V 16 h	5.208	-12.97 to 23.39	No
	P + O 16 h vs. P + V 16 h	-9.209	-27.39 to 8.97	No
	C 24 h vs. P 24 h	-1.618	-11.97 to 8.728	No
	C 24 h vs. P + E 24 h	-12.88	-23.22 to -2.531	Yes
	C 24 h vs. P + O 24 h	-2.778	-13.12 to 7.569	No
	C 24 h vs. P + V 24 h	-10.26	-20.61 to 0.08585	No
	P 24 h vs. P + E 24 h	-11.26	-21.61 to -0.9122	Yes
	P 24 h vs. P + O 24 h	-1.159	-11.51 to 9.188	No
	P 24 h vs. P + V 24 h	-8.643	-18.99 to 1.704	No
	P + E 24 h vs. P + O 24 h	10.1	-0.2469 to 20.45	No
	P + E 24 h vs. P + V 24 h	2.616	-7.73 to 12.96	No
	P + O 24 h vs. P + V 24 h	-7.483	-17.83 to 2.863	No
DG 18:0/18:0	C 4 h vs. P 4 h	-0.09587	-0.2272 to 0.03543	No
	C 4 h vs. P + E 4 h	-0.05137	-0.1827 to 0.07993	No
	C 4 h vs. P + O 4 h	-0.01614	-0.1474 to 0.1152	No
	C 4 h vs. P + V 4 h	-0.03538	-0.1667 to 0.09593	No
	P 4 h vs. P + E 4 h	0.0445	-0.0868 to 0.1758	No
	P 4 h vs. P + O 4 h	0.07974	-0.05157 to 0.211	No
	P 4 h vs. P + V 4 h	0.06049	-0.07081 to 0.1918	No
	P + E 4 h vs. P + O 4 h	0.03523	-0.09607 to 0.1665	No
	P + E 4 h vs. P + V 4 h	0.01599	-0.1153 to 0.1473	No
	P + O 4 h vs. P + V 4 h	-0.01924	-0.1505 to 0.1121	No
	C 8 h vs. P 8 h	-0.2904	-0.4465 to -0.1342	Yes
	C 8 h vs. P + E 8 h	-0.04091	-0.1971 to 0.1153	No
	C 8 h vs. P + O 8 h	-0.02601	-0.1822 to 0.1302	No
	C 8 h vs. P + V 8 h	-0.04944	-0.2056 to 0.1067	No
	P 8 h vs. P + E 8 h	0.2495	0.09331 to 0.4056	Yes
	P 8 h vs. P + O 8 h	0.2644	0.1082 to 0.4205	Yes
	P 8 h vs. P + V 8 h	0.2409	0.08478 to 0.3971	Yes
	P + E 8 h vs. P + O 8 h	0.0149	-0.1413 to 0.1711	No
	P + E 8 h vs. P + V 8 h	-0.008529	-0.1647 to 0.1476	No

	P + O 8 h vs. P + V 8 h	-0.02343	-0.1796 to 0.1327	No
	C 16 h vs. P 16 h	-0.3285	-0.6157 to -0.04139	Yes
	C 16 h vs. P + E 16 h	-0.121	-0.4082 to 0.1661	No
	C 16 h vs. P + O 16 h	-0.0031	-0.2902 to 0.284	No
	C 16 h vs. P + V 16 h	-0.1032	-0.3903 to 0.184	No
	P 16 h vs. P + E 16 h	0.2075	-0.07964 to 0.4946	No
	P 16 h vs. P + O 16 h	0.3254	0.03829 to 0.6126	Yes
	P 16 h vs. P + V 16 h	0.2254	-0.06176 to 0.5125	No
	P + E 16 h vs. P + O 16 h	0.1179	-0.1692 to 0.4051	No
	P + E 16 h vs. P + V 16 h	0.01788	-0.2693 to 0.305	No
	P + O 16 h vs. P + V 16 h	-0.1	-0.3872 to 0.1871	No
	C 24 h vs. P 24 h	-0.2365	-0.4405 to -0.03253	Yes
	C 24 h vs. P + E 24 h	-0.05092	-0.2549 to 0.1531	No
	C 24 h vs. P + O 24 h	-0.03484	-0.2388 to 0.1691	No
	C 24 h vs. P + V 24 h	0.002329	-0.2017 to 0.2063	No
	P 24 h vs. P + E 24 h	0.1856	-0.01839 to 0.3896	No
	P 24 h vs. P + O 24 h	0.2017	-0.002308 to 0.4057	No
	P 24 h vs. P + V 24 h	0.2388	0.03486 to 0.4428	Yes
	P + E 24 h vs. P + O 24 h	0.01608	-0.1879 to 0.2201	No
	P + E 24 h vs. P + V 24 h	0.05325	-0.1507 to 0.2572	No
	P + O 24 h vs. P + V 24 h	0.03717	-0.1668 to 0.2412	No
DG 18:0/18:1	C 4 h vs. P 4 h	-0.03258	-0.653 to 0.5879	No
	C 4 h vs. P + E 4 h	-0.2066	-0.8271 to 0.4139	No
	C 4 h vs. P + O 4 h	-0.2588	-0.8792 to 0.3617	No
	C 4 h vs. P + V 4 h	-0.2434	-0.8639 to 0.377	No
	P 4 h vs. P + E 4 h	-0.174	-0.7945 to 0.4464	No
	P 4 h vs. P + O 4 h	-0.2262	-0.8467 to 0.3943	No
	P 4 h vs. P + V 4 h	-0.2109	-0.8313 to 0.4096	No
	P + E 4 h vs. P + O 4 h	-0.05217	-0.6726 to 0.5683	No
	P + E 4 h vs. P + V 4 h	-0.03683	-0.6573 to 0.5836	No
	P + O 4 h vs. P + V 4 h	0.01534	-0.6051 to 0.6358	No
	C 8 h vs. P 8 h	-0.3816	-1.402 to 0.6389	No
	C 8 h vs. P + E 8 h	-0.3077	-1.328 to 0.7129	No
	C 8 h vs. P + O 8 h	-0.3857	-1.406 to 0.6348	No
	C 8 h vs. P + V 8 h	-0.3548	-1.375 to 0.6657	No
	P 8 h vs. P + E 8 h	0.0739	-0.9466 to 1.094	No
	P 8 h vs. P + O 8 h	-0.00418	-1.025 to 1.016	No
	P 8 h vs. P + V 8 h	0.02674	-0.9938 to 1.047	No
	P + E 8 h vs. P + O 8 h	-0.07808	-1.099 to 0.9424	No
	P + E 8 h vs. P + V 8 h	-0.04716	-1.068 to 0.9733	No
	P + O 8 h vs. P + V 8 h	0.03092	-0.9896 to 1.051	No
	C 16 h vs. P 16 h	-0.4493	-1.982 to 1.083	No
	C 16 h vs. P + E 16 h	-0.803	-2.336 to 0.7297	No
	C 16 h vs. P + O 16 h	-0.3912	-1.924 to 1.141	No
	C 16 h vs. P + V 16 h	-0.2733	-1.806 to 1.259	No
	P 16 h vs. P + E 16 h	-0.3536	-1.886 to 1.179	No
	P 16 h vs. P + O 16 h	0.05807	-1.475 to 1.591	No

	P 16 h vs. P + V 16 h	0.176	-1.357 to 1.709	No
	P + E 16 h vs. P + O 16 h	0.4117	-1.121 to 1.944	No
	P + E 16 h vs. P + V 16 h	0.5297	-1.003 to 2.062	No
	P + O 16 h vs. P + V 16 h	0.118	-1.415 to 1.651	No
	C 24 h vs. P 24 h	0.2131	-1.416 to 1.842	No
	C 24 h vs. P + E 24 h	-0.3471	-1.976 to 1.282	No
	C 24 h vs. P + O 24 h	-0.523	-2.152 to 1.106	No
	C 24 h vs. P + V 24 h	0.1587	-1.47 to 1.787	No
	P 24 h vs. P + E 24 h	-0.5603	-2.189 to 1.068	No
	P 24 h vs. P + O 24 h	-0.7362	-2.365 to 0.8925	No
	P 24 h vs. P + V 24 h	-0.05445	-1.683 to 1.574	No
	P + E 24 h vs. P + O 24 h	-0.1759	-1.805 to 1.453	No
	P + E 24 h vs. P + V 24 h	0.5058	-1.123 to 2.134	No
	P + O 24 h vs. P + V 24 h	0.6817	-0.9469 to 2.31	No
DG 18:1/18:1	C 4 h vs. P 4 h	0.1076	-1.609 to 1.825	No
	C 4 h vs. P + E 4 h	-1.035	-2.752 to 0.6819	No
	C 4 h vs. P + O 4 h	-1.411	-3.128 to 0.306	No
	C 4 h vs. P + V 4 h	-1.426	-3.144 to 0.2906	No
	P 4 h vs. P + E 4 h	-1.143	-2.86 to 0.5744	No
	P 4 h vs. P + O 4 h	-1.519	-3.236 to 0.1985	No
	P 4 h vs. P + V 4 h	-1.534	-3.251 to 0.183	No
	P + E 4 h vs. P + O 4 h	-0.3759	-2.093 to 1.341	No
	P + E 4 h vs. P + V 4 h	-0.3914	-2.108 to 1.326	No
	P + O 4 h vs. P + V 4 h	-0.01546	-1.733 to 1.702	No
	C 8 h vs. P 8 h	0.02024	-3.004 to 3.044	No
	C 8 h vs. P + E 8 h	-2.089	-5.114 to 0.9349	No
	C 8 h vs. P + O 8 h	-1.303	-4.327 to 1.721	No
	C 8 h vs. P + V 8 h	-2.363	-5.387 to 0.6617	No
	P 8 h vs. P + E 8 h	-2.11	-5.134 to 0.9147	No
	P 8 h vs. P + O 8 h	-1.323	-4.348 to 1.701	No
	P 8 h vs. P + V 8 h	-2.383	-5.407 to 0.6414	No
	P + E 8 h vs. P + O 8 h	0.7861	-2.238 to 3.81	No
	P + E 8 h vs. P + V 8 h	-0.2733	-3.298 to 2.751	No
	P + O 8 h vs. P + V 8 h	-1.059	-4.084 to 1.965	No
	C 16 h vs. P 16 h	-0.09445	-6.748 to 6.56	No
	C 16 h vs. P + E 16 h	-6.059	-12.71 to 0.5951	No
	C 16 h vs. P + O 16 h	-1.542	-8.196 to 5.112	No
	C 16 h vs. P + V 16 h	-2.558	-9.212 to 4.096	No
	P 16 h vs. P + E 16 h	-5.964	-12.62 to 0.6895	No
	P 16 h vs. P + O 16 h	-1.448	-8.102 to 5.206	No
	P 16 h vs. P + V 16 h	-2.464	-9.118 to 4.19	No
	P + E 16 h vs. P + O 16 h	4.517	-2.137 to 11.17	No
	P + E 16 h vs. P + V 16 h	3.501	-3.153 to 10.15	No
	P + O 16 h vs. P + V 16 h	-1.016	-7.67 to 5.638	No
	C 24 h vs. P 24 h	0.735	-3.144 to 4.614	No
	C 24 h vs. P + E 24 h	-4.474	-8.352 to -0.5946	Yes
	C 24 h vs. P + O 24 h	-1.121	-5 to 2.758	No

C 24 h vs. P + V 24 h	-1.906	-5.785 to 1.973	No
P 24 h vs. P + E 24 h	-5.209	-9.087 to -1.33	Yes
P 24 h vs. P + O 24 h	-1.856	-5.735 to 2.023	No
P 24 h vs. P + V 24 h	-2.641	-6.52 to 1.238	No
P + E 24 h vs. P + O 24 h	3.352	-0.5265 to 7.231	No
P + E 24 h vs. P + V 24 h	2.567	-1.311 to 6.446	No
P + O 24 h vs. P + V 24 h	-0.785	-4.664 to 3.094	No

Table S2. Statistical analysis of diglyceride contents. Data shown in Table 1 have been analyzed for statistically significant differences by using GraphPad Prism software (version 7) and the Tukey's multiple comparison test. Differences of $p < 0.05$ were considered as significant.

	**Control**		**Palmitate**		**Palmitate + Elaidate**		**Palmitate + Oleate**		**Palmitate + Vaccenate**	
	Mean	*SEM*	*Mean*	*SEM*	*Mean*	*SEM*	*Mean*	*SEM*	*Mean*	*SEM*
	ng/mg protein									
	4 h									
**Ceramide 16:0**	728.86	45.65	2308.87	159.12	1177.80	43.01	1293.94	64.33	1436.51	91.91
**Ceramide 18:0**	405.24	25.34	1210.12	96.92	478.24	29.57	536.89	27.88	480.08	37.03
**Ceramide 18:1**	7.92	1.01	11.79	1.42	319.50	8.25	23.38	1.87	471.20	39.01
	8 h									
**Ceramide 16:0**	735.59	16.59	3991.73	83.31	1421.84	74.99	1615.90	45.47	1907.96	22.83
**Ceramide 18:0**	383.83	14.27	2176.39	70.88	557.59	33.09	700.97	28.72	616.56	23.21
**Ceramide 18:1**	7.54	0.70	14.82	0.81	373.01	6.62	26.50	1.54	572.02	23.88
	16 h									
**Ceramide 16:0**	850.37	17.59	3904.21	116.47	1503.58	32.54	1419.29	63.50	1694.08	55.47
**Ceramide 18:0**	419.20	7.28	2044.46	276.45	615.47	31.12	584.42	36.99	597.56	24.40
**Ceramide 18:1**	10.65	0.97	13.06	1.56	381.25	6.90	19.82	1.52	464.19	31.05
	24 h									
**Ceramide 16:0**	802.35	16.53	3408.13	132.33	1494.63	30.99	1467.69	30.97	1704.50	24.28
**Ceramide 18:0**	415.71	13.17	2287.21	80.11	647.79	41.25	590.17	13.92	639.20	20.98
**Ceramide 18:1**	9.47	0.80	16.63	0.46	486.93	19.64	21.88	1.10	447.96	5.99

Table S3. Changes in ceramide levels. Cells were treated with BSA (control cells) or BSA-conjugated palmitate alone, or with palmitate and one of the unsaturated fatty acids, elaidate, oleate or vaccenate at 250 µM individual concentration for 8 h at 70-80% confluence. The amount of three different ceramide species was measured by LC-MS/MS in the washed cell samples prepared after 4, 8, 16 or 24 hour treatments. The detected ceramides contained either a palmitate (16:0) or a stearate (18:0) or a monounsaturated fatty acid of 18 carbons (18:1). Data were normalized to the protein content of the samples (ng/mg protein units), and are shown as mean values and SEM of three independent experiments with two parallels.

	Tukey's multiple comparisons test			
		**Mean Diff.**	**95.00% CI. of diff.**	**Significant?**
**Ceramide 16:0**	C 4 h vs. P 4 h	-1.58	-2.473 to -0.6868	Yes
	C 4 h vs. P + E 4 h	-0.4489	-1.342 to 0.4443	No
	C 4 h vs. P + O 4 h	-0.5651	-1.458 to 0.3282	No
	C 4 h vs. P + V 4 h	-0.7077	-1.601 to 0.1856	No
	P 4 h vs. P + E 4 h	1.131	0.2378 to 2.024	Yes
	P 4 h vs. P + O 4 h	1.015	0.1217 to 1.908	Yes
	P 4 h vs. P + V 4 h	0.8724	-0.02088 to 1.766	No
	P + E 4 h vs. P + O 4 h	-0.1161	-1.009 to 0.7771	No
	P + E 4 h vs. P + V 4 h	-0.2587	-1.152 to 0.6345	No
	P + O 4 h vs. P + V 4 h	-0.1426	-1.036 to 0.7507	No
	C 8 h vs. P 8 h	-3.256	-3.713 to -2.8	Yes
	C 8 h vs. P + E 8 h	-0.6863	-1.143 to -0.2297	Yes
	C 8 h vs. P + O 8 h	-0.8803	-1.337 to -0.4238	Yes
	C 8 h vs. P + V 8 h	-1.172	-1.629 to -0.7158	Yes
	P 8 h vs. P + E 8 h	2.57	2.113 to 3.026	Yes
	P 8 h vs. P + O 8 h	2.376	1.919 to 2.832	Yes
	P 8 h vs. P + V 8 h	2.084	1.627 to 2.54	Yes
	P + E 8 h vs. P + O 8 h	-0.1941	-0.6506 to 0.2625	No
	P + E 8 h vs. P + V 8 h	-0.4861	-0.9427 to -0.02957	Yes
	P + O 8 h vs. P + V 8 h	-0.2921	-0.7486 to 0.1645	No
	C 16 h vs. P 16 h	-3.054	-3.702 to -2.405	Yes
	C 16 h vs. P + E 16 h	-0.6532	-1.302 to -0.004838	Yes
	C 16 h vs. P + O 16 h	-0.5689	-1.217 to 0.07945	No
	C 16 h vs. P + V 16 h	-0.8437	-1.492 to -0.1953	Yes
	P 16 h vs. P + E 16 h	2.401	1.752 to 3.049	Yes
	P 16 h vs. P + O 16 h	2.485	1.837 to 3.133	Yes
	P 16 h vs. P + V 16 h	2.21	1.562 to 2.859	Yes
	P + E 16 h vs. P + O 16 h	0.08429	-0.5641 to 0.7327	No
	P + E 16 h vs. P + V 16 h	-0.1905	-0.8389 to 0.4579	No
	P + O 16 h vs. P + V 16 h	-0.2748	-0.9232 to 0.3736	No
	C 24 h vs. P 24 h	-2.606	-3.228 to -1.984	Yes
	C 24 h vs. P + E 24 h	-0.6923	-1.314 to -0.07019	Yes
	C 24 h vs. P + O 24 h	-0.6653	-1.287 to -0.04325	Yes
	C 24 h vs. P + V 24 h	-0.9021	-1.524 to -0.2801	Yes
	P 24 h vs. P + E 24 h	1.914	1.291 to 2.536	Yes
	P 24 h vs. P + O 24 h	1.94	1.318 to 2.563	Yes
	P 24 h vs. P + V 24 h	1.704	1.082 to 2.326	Yes
	P + E 24 h vs. P + O 24 h	0.02694	-0.5952 to 0.649	No
	P + E 24 h vs. P + V 24 h	-0.2099	-0.832 to 0.4122	No
	P + O 24 h vs. P + V 24 h	-0.2368	-0.8589 to 0.3853	No

**Ceramide 18:0**	C 4 h vs. P 4 h	-0.8049	-1.304 to -0.3059	Yes
	C 4 h vs. P + E 4 h	-0.073	-0.572 to 0.426	No
	C 4 h vs. P + O 4 h	-0.1317	-0.6307 to 0.3674	No
	C 4 h vs. P + V 4 h	-0.07483	-0.5739 to 0.4242	No
	P 4 h vs. P + E 4 h	0.7319	0.2329 to 1.231	Yes
	P 4 h vs. P + O 4 h	0.6732	0.1742 to 1.172	Yes
	P 4 h vs. P + V 4 h	0.73	0.231 to 1.229	Yes
	P + E 4 h vs. P + O 4 h	-0.05865	-0.5577 to 0.4404	No
	P + E 4 h vs. P + V 4 h	-0.001831	-0.5009 to 0.4972	No
	P + O 4 h vs. P + V 4 h	0.05682	-0.4422 to 0.5558	No
	C 8 h vs. P 8 h	-1.793	-2.175 to -1.41	Yes
	C 8 h vs. P + E 8 h	-0.1738	-0.5566 to 0.2091	No
	C 8 h vs. P + O 8 h	-0.3171	-0.7 to 0.06571	No
	C 8 h vs. P + V 8 h	-0.2327	-0.6156 to 0.1501	No
	P 8 h vs. P + E 8 h	1.619	1.236 to 2.002	Yes
	P 8 h vs. P + O 8 h	1.475	1.093 to 1.858	Yes
	P 8 h vs. P + V 8 h	1.56	1.177 to 1.943	Yes
	P + E 8 h vs. P + O 8 h	-0.1434	-0.5262 to 0.2395	No
	P + E 8 h vs. P + V 8 h	-0.05897	-0.4418 to 0.3239	No
	P + O 8 h vs. P + V 8 h	0.0844	-0.2984 to 0.4672	No
	C 16 h vs. P 16 h	-1.625	-2.856 to -0.3947	Yes
	C 16 h vs. P + E 16 h	-0.1963	-1.427 to 1.034	No
	C 16 h vs. P + O 16 h	-0.1652	-1.396 to 1.065	No
	C 16 h vs. P + V 16 h	-0.1784	-1.409 to 1.052	No
	P 16 h vs. P + E 16 h	1.429	0.1984 to 2.66	Yes
	P 16 h vs. P + O 16 h	1.46	0.2295 to 2.691	Yes
	P 16 h vs. P + V 16 h	1.447	0.2163 to 2.677	Yes
	P + E 16 h vs. P + O 16 h	0.03104	-1.2 to 1.262	No
	P + E 16 h vs. P + V 16 h	0.0179	-1.213 to 1.248	No
	P + O 16 h vs. P + V 16 h	-0.01314	-1.244 to 1.217	No
	C 24 h vs. P 24 h	-1.872	-2.284 to -1.459	Yes
	C 24 h vs. P + E 24 h	-0.2321	-0.6447 to 0.1805	No
	C 24 h vs. P + O 24 h	-0.1745	-0.5871 to 0.2381	No
	C 24 h vs. P + V 24 h	-0.2235	-0.6361 to 0.1891	No
	P 24 h vs. P + E 24 h	1.639	1.227 to 2.052	Yes
	P 24 h vs. P + O 24 h	1.697	1.284 to 2.11	Yes
	P 24 h vs. P + V 24 h	1.648	1.235 to 2.061	Yes
	P + E 24 h vs. P + O 24 h	0.05762	-0.355 to 0.4702	No
	P + E 24 h vs. P + V 24 h	0.008594	-0.404 to 0.4212	No
	P + O 24 h vs. P + V 24 h	-0.04902	-0.4616 to 0.3636	No
**Ceramide 18:1**	C 4 h vs. P 4 h	-0.003869	-0.1784 to 0.1706	No
	C 4 h vs. P + E 4 h	-0.3116	-0.4861 to -0.1371	Yes
	C 4 h vs. P + O 4 h	-0.01546	-0.19 to 0.159	No
	C 4 h vs. P + V 4 h	-0.4633	-0.6378 to -0.2888	Yes
	P 4 h vs. P + E 4 h	-0.3077	-0.4822 to -0.1332	Yes
	P 4 h vs. P + O 4 h	-0.01159	-0.1861 to 0.1629	No

P 4 h vs. P + V 4 h	-0.4594	-0.6339 to -0.2849	Yes
P + E 4 h vs. P + O 4 h	0.2961	0.1216 to 0.4706	Yes
P + E 4 h vs. P + V 4 h	-0.1517	-0.3262 to 0.0228	No
P + O 4 h vs. P + V 4 h	-0.4478	-0.6223 to -0.2733	Yes
C 8 h vs. P 8 h	-0.007279	-0.1158 to 0.1012	No
C 8 h vs. P + E 8 h	-0.3655	-0.474 to -0.257	Yes
C 8 h vs. P + O 8 h	-0.01896	-0.1275 to 0.08956	No
C 8 h vs. P + V 8 h	-0.5645	-0.673 to -0.456	Yes
P 8 h vs. P + E 8 h	-0.3582	-0.4667 to -0.2497	Yes
P 8 h vs. P + O 8 h	-0.01168	-0.1202 to 0.09684	No
P 8 h vs. P + V 8 h	-0.5572	-0.6657 to -0.4487	Yes
P + E 8 h vs. P + O 8 h	0.3465	0.238 to 0.455	Yes
P + E 8 h vs. P + V 8 h	-0.199	-0.3075 to -0.09049	Yes
P + O 8 h vs. P + V 8 h	-0.5455	-0.654 to -0.437	Yes
C 16 h vs. P 16 h	-0.002409	-0.1417 to 0.1369	No
C 16 h vs. P + E 16 h	-0.3706	-0.5099 to -0.2313	Yes
C 16 h vs. P + O 16 h	-0.009163	-0.1484 to 0.1301	No
C 16 h vs. P + V 16 h	-0.4535	-0.5928 to -0.3143	Yes
P 16 h vs. P + E 16 h	-0.3682	-0.5075 to -0.2289	Yes
P 16 h vs. P + O 16 h	-0.006754	-0.146 to 0.1325	No
P 16 h vs. P + V 16 h	-0.4511	-0.5904 to -0.3119	Yes
P + E 16 h vs. P + O 16 h	0.3614	0.2222 to 0.5007	Yes
P + E 16 h vs. P + V 16 h	-0.08294	-0.2222 to 0.05633	No
P + O 16 h vs. P + V 16 h	-0.4444	-0.5836 to -0.3051	Yes
C 24 h vs. P 24 h	-0.007158	-0.09704 to 0.08272	No
C 24 h vs. P + E 24 h	-0.4775	-0.5673 to -0.3876	Yes
C 24 h vs. P + O 24 h	-0.01241	-0.1023 to 0.07747	No
C 24 h vs. P + V 24 h	-0.4385	-0.5284 to -0.3486	Yes
P 24 h vs. P + E 24 h	-0.4703	-0.5602 to -0.3804	Yes
P 24 h vs. P + O 24 h	-0.00525	-0.09513 to 0.08463	No
P 24 h vs. P + V 24 h	-0.4313	-0.5212 to -0.3415	Yes
P + E 24 h vs. P + O 24 h	0.4651	0.3752 to 0.5549	Yes
P + E 24 h vs. P + V 24 h	0.03897	-0.05091 to 0.1289	No
P + O 24 h vs. P + V 24 h	-0.4261	-0.516 to -0.3362	Yes

Table S4. Statistical analysis of ceramide levels. Data shown in Table 3 have been analyzed for statistically significant differences by using GraphPad Prism software (version 7) and the Tukey's multiple comparison test. Differences of $p < 0.05$ were considered as significant.
